# Dietary diversity score is associated with cardiovascular risk factors and serum adiponectin concentrations in patients with metabolic syndrome

**DOI:** 10.1186/s12872-018-0807-3

**Published:** 2018-04-17

**Authors:** Mahdieh Abbasalizad Farhangi, Leila Jahangiry

**Affiliations:** 1 Drug Applied Research Center, Nutrition Research Center, Tabriz University of Medical Sciences, Tabriz, Iran; 2 Nutrition Research Center, Tabriz University of Medical Sciences, Tabriz, Iran; 3 Tabriz Health Services Management Research Center, Health Education and Health Promotion Department, School of Public Health, Tehran University of Medical Sciences, Tehran, Iran

**Keywords:** Dietary diversity score, Metabolic syndrome, Adiponectin

## Abstract

**Background:**

Metabolic syndrome is associated with cardio-metabolic risk factors and lipid abnormalities. Previous studies evaluated the dietary habits and nutrient intakes among patients with metabolic syndrome; however the association between metabolic risk factors and adiponectin with dietary diversity score (DDS) in patients with metabolic syndrome has not been evaluated yet. Therefore the aim of the current study was to evaluate these relationships among patients with metabolic syndrome.

**Methods:**

One hundred sixty patients with metabolic syndrome were recruited in the study. The anthropometric parameters including weight, height, waist circumference and hip circumference were measured. Serum adiponectin concentration was measured by enzyme- linked immunosorbent assay method (ELISA). Lipid profile and fasting serum glucose concentrations (FSG) were also measured with enzymatic colorimetric methods. Blood pressure was also measured and DDS was calculated using the data obtained from food frequency questionnaire (FFQ).

**Results:**

Subjects in lower DDS categorizes had significantly lower energy and fiber intake; whereas mean protein intake of subjects in the highest quartile was significantly higher than second quartile. Higher prevalence of obesity was also observed in the top quartiles (*P* < 0.001). Subjects in the lower quartiles had higher serum triglyceride concentrations and systolic blood pressure (SBP) values and lower serum adiponectin concentrations compared with subjects in higher DDS categorizes (*P* < 0.05). The prevalence of metabolic syndrome components among patients in lower DDS quartiles was significantly higher (P < 0.05).

**Conclusion:**

Our study found a lower serum triglyceride and SBP and higher serum adiponectin concentrations in top quartiles of DDS. The findings clarify the possible preventive role of higher dietary diversity score against metabolic syndrome. However, for further confirming the findings, more studies are warranted.

## Backgrounds

Metabolic syndrome first defined by Gerald Reaven [1] is a cluster of cardio-metabolic risk factors like hypertension, lipid abnormalities, insulin resistance and central obesity. The disease is associated with several abnormalities including cardiovascular risk factors [2], chronic kidney disease [3], non- alcoholic fatty liver disease [4] depression [5] and psychiatric disorders [6]. It is associated with a 2-fold increase in risk of cardiovascular disease (CVD), CVD mortality, and stroke, and a 1.5-fold increase in risk of all-cause mortality [7–9]. The prevalence of metabolic syndrome is increasing worldwide because of underlying increase in prevalence of obesity; the Third National Health and Nutrition Examination Survey (NHANES III) reported an alarming unadjusted and age-adjusted prevalence of the metabolic syndrome of 21.8% and 23.7%, respectively [10, 11]. In Iran according to the results of Tehran Lipid and Glucose Study (TLGS), 33.7% of adults aged ≥20 years old were suffering from metabolic syndrome [12]. Other studies reported 55% and 30.1% for the prevalence of metabolic syndrome in Iranian adult women and men respectively [13–15]. Complications of metabolic syndrome might be preventable by a healthy diet and dietary factors are most common environmental cause of the metabolic syndrome [16].

Numerous studies have evaluated the effects of single dietary components on the features of metabolic syndrome [17, 18]; however assessing overall diet instead of a single nutrient on diet-disease relations may be more informative. Dietary diversity score (DDS) is considered as a useful dietary approach to evaluate the total diet quality and correlates positively with nutritional adequacy [19]. Several previous studies evaluated the relationship between DDS and cardiovascular risk factors among patients with non-alcoholic fatty liver disease [20, 21]. The relationship between DDS and features of metabolic syndrome has also been evaluated by Azadbakht et al. [22] among healthy subjects aged over 18 years old reporting an inverse relation between components of metabolic syndrome and DDS.

Adiponectin, an adipose tissue derived polypeptide, is exclusively expressed in the white adipose tissue and to some extent in brown adipose tissue; it has insulin-sensitizing and anti-diabetic properties and is inversely associated with adiposity [23]; because of the close relationship between serum adiponectin and insulin resistance, it has also been proposed as a useful diagnostic criterion for diabetes or metabolic syndrome [24]. Because of the metabolic effects of adiponectin, one can hypothesize that it might be related to DDS score; however no reports assessing this relationship exist. Therefore in the current study, we evaluate the relationship between DDS, serum adiponectin and components of metabolic syndrome.

## Methods

### Subjects

The protocol of the current study has been explained before [25, 26]; briefly, one hundred and sixty patients with metabolic syndrome were selected according to the National Cholesterol Education Program’s Adult Treatment Panel III (NCEP-ATP III) criteria [12]. Subjects with the history of cardiovascular diseases, cancer, type 2 diabetes mellitus, renal diseases, being pregnant, taking medications for hypertension or dyslipidemia were excluded from the study. At the beginning of the study the subjects underwent a physical examination in which the information about weight, height, waist circumference (WC), hip circumference were obtained and waist to hip ratio (WHR), body mass index (BMI) were calculated. Overweight and obesity were defined as having BMI 25–29.9 kg/m^2^ and BMI > 30 kg/m^2^ respectively [27].

### Biochemical assays

Serum fating blood glucose (FSG), triglyceride (TG), aspartate aminotransferase (AST), alanine aminotransferase (ALT), total cholesterol (TC), high density lipoprotein cholesterol (HDL-C) and low density lipoprotein cholesterol (LDL) were analyzed by enzymatic colorimetric method (Pars – Azmoon, Tehran – Iran). Serum insulin was also analyzed with enzyme linked immunosorbent assay method (ELISA-Monobind Insulin AccuBind, CA 92630, USA); the sensitivity of this assay was 0.75 μIU/ml and mean inter and intra assay coefficient of variations (CV) were of < 9.8% and < 8% respectively. Serum adiponectin was analyzed by ELISA method (AviBion, Fin-01720 Vantaa, Finland) with sensitivity of < 0.185 ng/ml and mean inter and intra assay CV of ≤12% and ≤ 10% respectively.

### Dietary diversity score (DDS)

Usual dietary intake was assessed using a 147 item semi-quantitative standard food frequency questionnaire (FFQ) which was developed and validated in Tehran [28]. This FFQ consisted of a list of foods with standard serving sizes commonly used by Iranians. Participants reported frequency of consumption of a given food item during the previous year. Portion sizes of foods were converted to grams. Dietary diversity score was calculated by the method first developed by Kant et al. [19, 29]. Five food groups including bread and grains, vegetables, fruits, meats and dairy were considered according to the food groups introduced by US Department of Agriculture (USDA) food guide pyramid. These food groups were divided into 23 subgroups. Bread and grain group was divided into seven subgroups (refined bread, biscuits, macaroni, whole bread, corn flakes, rice and refined flour), vegetable group divided into seven subgroups (vegetable, potato, tomato, other starchy vegetables, legumes, yellow vegetables, green vegetables) and fruits into two subgroups (fruits and fruit juices). Four subgroups were defined for meat group (red meat, poultry, fish and eggs) and three subgroups were considered for dairy group (milk, yogurt and cheese). To be counted as a consumer of one food group, one should consume one-half serving in one day as defined by the food guide pyramid quantity criteria. The maximum score of 2 was considered for each food group. Therefore the total DDS was in the range of 0 to 10 [22].

### Statistical analysis

Data were analyzed using Statistical Package for Social Science (SPSS) version 18. Data were presented as mean ± SD or number and percentages. Cut of points for DDS scores were defined by quartiles categorizes. Comparison of variables between different DDS quartiles was performed by one-way ANOVA. Bonferroni correction test was used as *Post-Hoc* analysis. The comparison of qualitative variables distribution of across quartile categories of DDS was performed by χ ^2^ test. *P* values less than 0.05 were defined as the significance threshold. Multiple logistic regression model was used to evaluate the relationship with overweight and obesity with dietary diversity scores adjusted for confounder effects of age.

## Results

### General characteristics of study participants

The mean DDS of participants was 4.71 ± 1.16. Table 1 represents the comparison of demographic parameters, anthropometric variables and main food groups DDS across quartile categorizes of DDS. Mean age and gender distribution were not significantly different between quartiles of DDS; however persons in the lower DDS quartile categorize had significantly lower BMI (*P* < 0.05). High prevalence of obesity was also observed in highest quartiles (*P* < 0.001). Higher DDS score was also associated with higher educational attainment (*P* = 0.016).

**Table 1:** Characteristics of study population by quartiles of dietary diversity score (DDS)

Quartiles of DDS				
	1st (*n* = 37)	2nd (*n* = 34)	3rd (*n* = 46)	4th (*n* = 43)
Age (years)	46.14 ± 9.27	42.45 ± 9.63	43.81 ± 9.91	43.22 ± 11.40
Men [n (%)]	67.6%	58.1%	62.8%	70%
BMI (kg/m^2^)	29.05 ± 4.90^a^	29.33 ± 3.81	30.53 ± 4.90	31.59 ± 4.54
WC (cm)	103.36 ± 9.67	102.00 ± 7.68^b^	104.86 ± 8.16	106.83 ± 7.69
WHR	0.94 ± 4.90	0.90 ± 0.08	0.92 ± 0.05	0.95 ± 0.08
Obesity (%)^c^	15.9	17.4	29	37.7
Educational attainment ^d^				
*< 12 years*	8.6%	21.9%	2.3%	5%
*12*	45.7%	34.4%	41.9%	25%
*> 12 years*	45.7%	43.8%	55.8%	70%
Smoking [n (%)]	17.61	12.90	7	3
Grain diversity score	0.67 ± 0.15^e^	0.70 ± 0.21	0.83 ± 0.17	0.90 ± 0.26
Fruits diversity score	0.65 ± 0.48^e^	1.12 ± 0.33	1.23 ± 0.43	1.50 ± 0.51
Vegetable diversity score	0.48 ± 0.28^e^	0.68 ± 0.28	0.77 ± 0.32	0.96 ± 0.31
Dairy diversity score	1.16 ± 0.37^e^	1.66 ± 0.50	1.70 ± 0.36	1.85 ± 0.28
Meat diversity score	0.21 ± 0.34^e^	0.25 ± 0.13	0.39 ± 0.14	0.81 ± 0.43
Energy density (ED)	0.92 ± 0.25^e^	0.89 ± 0.18	0.87 ± 0.18	0.90 ± 0.24

*BMI* body mass index, *WC* waist circumference, *WHR* waist to hip ratio; ^a,b^ *P* < 0.05 compared with other quartiles *P* < 0.001; ^c,d^ χ^2^ = 27.62, *P* < 0.001 and χ^2^ = 14.62, *P* = 0.016 respectively; ^e^ *P* < 0.001 compared with other quartiles

### Dietary intakes and DDS

DDS of all of food groups in first quartile was lower than other quartiles. The mean energy density of first quartile of DDS was also significantly lower than other quartiles (Table 1). The comparison of mean energy, macronutrients and dietary fiber intake across different categorizes of DDS are presented in Table 2. Subjects in lower DDS categorizes had significantly lower energy and fiber intake (*P* < 0.001); whereas mean protein intake of subjects in the highest quartile was significantly higher than second quartile. Moreover, in binary logistic regression model, grain and dairy diversity score were significant predictors of obesity or overweight (Table 3).

**Table 2:** Dietary intakes of participants according to DDS quartile

Quartiles of DDS				
	1st (n = 37)	2nd (n = 34)	3rd (*n* = 46)	4th (n = 43)
Total energy (kcal/d)	2222.89 ± 94.73 ^a^	2826.19 ± 806.10	3086.56 ± 165.43	3668.98 ± 209.08
Carbohydrate (%)	62.97 ± 7.44	67.47 ± 5.55	61.50 ± 7.53	60.44 ± 5.71
Protein (%)	13.89 ± 2.54	12.32 ± 1.45	13.47 ± 2.36	15.28 ± 2.50^b^
Fat (%)	26.18 ± 5.44	24.91 ± 4.63	28.56 ± 6.53	27.83 ± 4.12
Dietary fiber (g/d)	45.58 ± 14.98^c^	82.74 ± 52.34	58.82 ± 19.11	60.78 ± 22.52

^a^ *P* < 0.001 compared with other quartiles; *P* = 0.033 compared with second quartile; ^c^ *P* < 0.001 compared with second quartile

**Table 3:** Binary logistic regression model with being overweight or obese as dependent variable and total or food groups DDS as independent variables

Independent variables	*β*	*S.E. β*	Wald’s χ^2^ (df = 1)	P	Odds Ratio
Grains diversity score	3.21	1.78	3.46	*0.05*	27.29
Fruit diversity score	−0.26	0.52	0.25	0.64	0.78
Vegetable diversity score	−0.17	0.95	0.03	0.78	0.87
Dairy diversity score	1.44	0.63	5.14	*0.022*	4.38
Meat diversity score	0.86	0.84	1.05	0.29	2.42

Nagelkerke’s *R*^2^ = 0.152, after adjustment for confounder effect of age and gender

The italic values are statistically significant

### Metabolic parameters and DDS

**Fig. 1:**
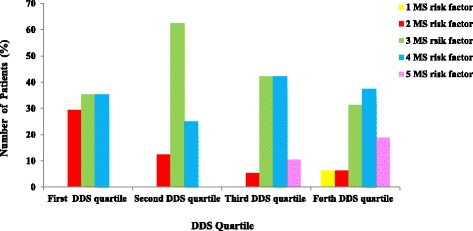
Association between DDS quartiles and number of metabolic syndrome components in patients (MS, Metabolic Syndrome; DDS, Dietary Diversity Score, *P*-value from Chi-Square test: 0.046)

Top quartiles of DDS score was also associated with better metabolic status (Table 4); subjects in lower quartiles had higher serum triglyceride concentrations and SBP values and lower serum adiponectin concentrations compared with subjects in higher DDS categorizes (*P* < 0.05). Serum HDL concentrations in the highest quartile was also higher than other quartiles however this difference did not achieve to significant threshold. The prevalence of components of metabolic syndrome in different DDS quartiles is presented in Fig. 1. As shown in this figure, patients in higher quartiles of DDS had significantly higher number of metabolic syndrome components (*P* = 0.04).

**Table 4:** Metabolic biomarkers of participant according to DDS quartile

Quartiles of DDS				
	1st (n = 37)	2nd (n = 34)	3rd (n = 46)	4th (n = 43)
FSG (mg/dl)	93.73 ± 12.40	88.00 ± 12.11	87.83 ± 13.90	90.55 ± 16.51
TG (mg/dl)	200.47 ± 104.85 ^a^	197.87 ± 141.02	194.76 ± 119.80	168.50 ± 71.25
TC (mg/dl)	189.29 ± 52.61	191.61 ± 38.11	187.34 ± 33.62	191.17 ± 37.63
LDL (mg/dl)	122.23 ± 4.56	123.12 ± 34.65	122.56 ± 45.98	122.18 ± 54.65
HDL (mg/dl)	40.05 ± 11.05	40.85 ± 9.36	41.30 ± 9.22	42.32 ± 10.78
SBP (mmHg)	133.05 ± 9.70^a^	132.08 ± 8.70	128.70 ± 8.56	128.69 ± 3.12
DBP (mmHg)	88.08 ± 5.07	87.74 ± 3.37	89.39 ± 6.65	87.62 ± 9.19
Adiponectin (ng/ml)	11.82 ± 4.35 ^a^	14.50 ± 4.12	14.27 ± 3.70	15.27 ± 5.72

^a^ *P* < 0.001 compared with other quartiles; *FSG* fasting serum glucose, *TG* triglyceride, *TC* total cholesterol, *LDL* low density lipoprotein, *HDL* high density lipoprotein, *SBP* systolic blood pressure, *DBP* diastolic blood pressure, *HOMA-IR* homeostatic model assessment for insulin resistance, *AST* aspartate aminotransferase, *ALT* alanine aminotransferase

## Discussion

The current study conducted among patients with metabolic syndrome and showed that higher dietary diversity score is associated with healthy dietary habits and better metabolic features. Patients in lower DDS scores had significantly higher serum triglyceride and SBP values and lower adiponectin concentrations compared with patients in other quartiles. To our review of literature there was only one study evaluated the relationship between dietary diversity scores and metabolic syndrome in Iranian healthy population however our study is the first one evaluated such a relationship in patients with metabolic syndrome. Furth more we also evaluated the relationship between DDS with serum adiponectin which has not been evaluated before.

The results of the current study showed that increase in energy and macronutrients intake was associated with increase in DDS scores; these findings was in consistent with the finding of the study by Azadbakht et al. [30] reporting a positive association between DDS and energy intakes among female students. Several other studies also found similar results [31, 32]. Additionally, higher DDS was also associated with higher BMI and higher prevalence of obesity. It was not unexpected; since eating a more varied diet is associated with higher intakes of energy and macronutrients and also higher nutritional adequacy [33]. In our study higher energy intake in higher DDS was attributed to higher consumption of protein and fiber not fat or carbohydrate. Previous reports by Azadbakht et al. found similar results [30, 34]. Interestingly these reports were similar to our findings reporting higher BMI and higher prevalence of obesity in higher DDS scores; they suggested that higher energy intakes in their study was attributed to higher intakes of healthy foods including fruits, vegetables and whole grains rather than meat or refined grains; however we found higher intakes of almost all of food groups in our study. These inconsistencies might be attributed to the study samples as we studied patients with metabolic syndrome rather than total population. Dairy consumption diversity score alongside with grain diversity score were potent predictors of obesity in the current research; although several previous researches had reported an inverse association between calcium intake and body mass index or obesity [35, 36], however the fat content of the dairy products might attenuate the beneficial effects of its calcium content.

In our study higher DDS scores were associated with lower serum triglyceride and systolic blood pressure and higher serum adiponectin concentrations. These findings were in consistent with previous studies by Azadbakht et al. reporting lower probability of hypertension, hypercholesterolemia, diabetes and high LDL concentrations in higher DDS categorizes in Tehranian adults [22]. In other report similar to our results higher DDS was significantly associated with lower risk of hypertension and hypertriglyceridemia [34]. Previous study by Kant et al. [19] found an inverse association between age-adjusted risk of mortality and DDS (*P* < 0.0009) in men and women. Diets with higher varieties are associated with increased intakes of essential micronutrients including vitamin C, calcium and also dietary fiber intake [37]. Our study for the first time reported higher serum adiponectin concentrations in higher DDS categorizes; previous studies have established positive association between healthy dietary parameters and serum adiponectin concentrations [38, 39]. In the study by Ostrowska L et al. [40], serum adiponectin concentrations was positively associated with dietary intakes of vegetable, vegetable oil, coffee and tea whilst a negative correlation was seen with consumptions of mixed bread, fried and baked dishes, alcohol, nuts and seeds. Other studies also confirmed the positive associations between serum adiponectin concentrations and frequency of green tea consumption [41]. Consistent with these findings our study also found that higher DDS, indicating healthier dietary habits, are associated with higher serum adiponectin concentrations.

## Conclusion

Our study showed that higher DDS was associated with better metabolic features and higher serum adiponectin concentrations; although higher BMI was also found in higher DDS categorizes. The cross-sectional design of the current study makes us unable to further evaluate the relationship between variables; further studies with case control or interventional designs are needed to confirm our findings.

## Abbreviations

ANOVA: analysis of Variance
BMI: body mass index
CAD: coronary artery disease
CRP: C-reactive protein
CVD: cardiovascular disease
DBP: diastolic blood pressure
DDS: dietary diversity score
FSG: fasting serum glucose
HDL: high density lipoprotein
LDL: low density lipoprotein
NCEP-ATP: National Cholesterol Education Adult Treatment Panel
SBP: systolic blood pressure
TC: total cholesterol
TG: triglyceride
USDA: United States’ department of agriculture
WC: waist circumference

## References

[1] Reaven GM (1988). Role of insulin resistance in human disease. Diabetes.

[2] Isomaa B, Almgren P, Tuomi T, Forsén B, Lahti K, Nissén M (2001). Cardiovascular morbidity and mortality associated with the metabolic syndrome. Diabetes Care.

[3] Ritz E (2008). Metabolic syndrome and kidney disease. Blood Purif.

[4] Marchesini G, Brizi M, Bianchi G, Tomassetti S, Bugianesi E, Lenzi M (2001). Nonalcoholic fatty liver disease a feature of the metabolic syndrome. Diabetes.

[5] Heiskanen TH, Niskanen LK, Hintikka JJ, Koivumaa-Honkanen HT, Honkalampi KM, Haatainen KM (2006). Metabolic syndrome and depression: a cross-sectional analysis. J Clin Psychiatry.

[6] Singh S, Mattoo S (2008). Metabolic syndrome and psychiatric disorders. Indian J Med Res.

[7] Mottillo S, Filion KB, Genest J, Joseph L, Pilote L, Poirier P, et al. The metabolic syndrome and cardiovascular risk: a systematic review and meta-analysis. J Am Coll Cardiol2010;56(14):1113–1132. 10.1016/j.jacc.2010.05.034 20863953

[8] Sokhanvar S, Khoshi A, Hajiaghaei S, Mousavinasab SN, Golmohammadi Z (2012). Association between Apo-lipoprotein-B levels at admission of patients and short-term morbidity and mortality after myocardial infarction. J Cardiovasc Thorac Res.

[9] Aggarwal A, Aggarwal S, Sharma V (2014). Cardiovascular risk factors in young patients of coronary artery disease: differences over a decade. J Cardiovasc Thorac Res.

[10] Ford ES, Giles WH, Dietz WH (2002). Prevalence of the metabolic syndrome among US adults: findings from the third National Health and nutrition examination survey. JAMA.

[11] Heshmat R, Shafiee G, Qorbani M, Azizi-Soleiman F, Djalalinia SH, Motlagh ME (2016). Association of ghrelin with cardiometabolic risk factors in Iranian adolescents: the CASPIAN-III study. J Cardiovasc Thorac Res.

[12] Zabetian A, Hadaegh F, Azizi F. Prevalence of metabolic syndrome in Iranian adult population, concordance between the IDF with the ATP III and the WHO definitions. Diabetes res Clin Pract. Diabetes Res Clin Pract 2007;77 (2):251–257. 10.1016/j.diabres.2006.12.001 17234299

[13] Azimi-Nezhad M, Herbeth B, Siest G, Dade S, Ndiaye NC, Esmaily H (2012). High prevalence of metabolic syndrome in Iran in comparison with France: what are the components that explain this?. Metab Syndr Relat Disord.

[14] Shafayi FS, Akef M, Sadegi H, Niknazhad AS (2012). A comparison of physical activity and nutritional practices in hypertensive and non- hypertensive pregnant women. J Cardiovasc Thorac Res.

[15] Sharifi N, Mahdavi R, Ebrahimi-Mameghani M (2013). Perceived barriers to weight loss programs for overweight or obese women. Health Prom Perspect.

[16] Lutsey PL, Steffen LM, Stevens J (2008). Dietary intake and the development of the metabolic syndrome: the atherosclerosis risk in communities study. Circulation.

[17] McKeown NM, Meigs JB, Liu S, Saltzman E, PWF W, Jacques PF (2004). Carbohydrate nutrition, insulin resistance, and the prevalence of the metabolic syndrome in the Framingham offspring cohort. Diabetes Care.

[18] Esposito K, Marfella R, Ciotola M, Di Palo C, Giugliano F, Giugliano G (2004). Effect of a Mediterranean-style diet on endothelial dysfunction and markers of vascular inflammation in the metabolic syndrome: a randomized trial. JAMA.

[19] Kant AK, Schatzkin A, Harris TB, Ziegler RG, Block G (1993). Dietary diversity and subsequent mortality in the first National Health and nutrition examination survey epidemiologic follow-up study. The AmJClinNutr.

[20] Toft U, Kristoffersen L, Lau C, Borch-Johnsen K, Jorgensen T (2006). The dietary quality score: validation and association with cardiovascular risk factors: the Inter99 study. Eur JClinNutr.

[21] Alavian SM, Esmaillzadeh A, Adibi P, Azadbakht L (2013). Dietary quality indices and biochemical parameters among patients with non alcoholic fatty liver disease (NAFLD). Hepat Mon.

[22] Azadbakht L, Mirmiran P, Esmaillzadeh A, Azizi F (2006). Dietary diversity score and cardiovascular risk factors in Tehranian adults. Public HealthNutr.

[23] Meilleur K, Doumatey A, Huang H, Charles B, Chen G, Zhou J (2010). Circulating adiponectin is associated with obesity and serum lipids in west Africans. J Clin Endocrinol Metab.

[24] Mojiminiyi OA, Abdella NA, Arouj MA, Nakhi AB (2007). Adiponectin, insulin resistance and clinical expression of the metabolic syndrome in patients with type 2 diabetes. Int J Obes.

[25] Farhangi MA, Jahangiry L, Mirinazhad MM, Shojaeezade D, Montazeri A, Yaghoubi A (2017). A web-based interactive lifestyle modification program improves lipid profile and serum adiponectin concentrations in patients with metabolic syndrome: the “red ruby” study. Int J Diab Develop Ctrs.

[26] Farhangi MA, Jahangiry L, Asghari-Jafarabadi M, Najafi M (2016). Association between dietary patterns and metabolic syndrome in a sample of Tehranian adults. Obes Res Clin Pract.

[27] Farhangi MA, Keshavarz SA, Eshraghian M, Ostadrahimi A, Saboor-Yaraghi AA (2013). Vitamin a supplementation, serum lipids, liver enzymes and C-reactive protein concentrations in obese women of reproductive age. Ann Clin Biochem.

[28] Hosseini-Esfahani F, Asghari G, Mirmiran P (2010). Reproducibility and relative validity of food group intake in a food frequency questionnaire developed for the Tehran lipid and glucose study. J Epidemiol.

[29] Kant AK, Block G, Schatzkin A, Ziegler RG, Nestle M (1991). Dietary diversity in the US population, NHANES II, 1976-1980. J Am Diet Assoc.

[30] Azadbakht L, Esmaillzadeh A (2010). Dietary diversity score is related to obesity and abdominal adiposity among Iranian female youth. Public Health Nutr.

[31] Raynor HA, Epstein LH (2001). Dietary variety, energy regulation, and obesity. Psychol Bull.

[32] Raynor HA, Jeffery RW, Tate DF (2004). Relationship between changes in food group variety, dietary intake, and weight during obesity treatment. Int J Obes Relat Metab Disord.

[33] Steyn N, Nel J, Nantel G, Kennedy G, Labadarios D (2006). Food variety and dietary diversity scores; are they good indicators of dietary adequacy?. Public Health Nutr.

[34] Azadbakht L, Mirmiran P, Azizi F (2005). Dietary diversity score is favorably associated with the metabolic syndrome in Tehranian adults. Int J Obes.

[35] Jacobsen R, Lorenzen JK, Toubro S, Krog-Mikkelsen I, Astrup A (2005). Effect of short-term high dietary calcium intake on 24-h energy expenditure, fat oxidation, and fecal fat excretion. Int J Obes Relat Metab Disord.

[36] Schrager S (2005). Dietary calcium intake and obesity. JABFP.

[37] Mirmiran P, Azadbakht L, Esmaillzadeh A, Azizi F (2004). Dietary diversity score in adolescents – a good indicator of the nutritional adequacy of diets: Tehran lipid and glucose study. Asia Pac J Clin Nutr.

[38] Pischon T, Girman CJ, Rifai N, Hotamisligil GS, Rimm EB (2005). Association between dietary factors and plasma adiponectin concentrations in men. Am JClin Nutr.

[39] Nakamura Y, Ueshima H, Okuda N, Higashiyama A, Kita Y, Kadowaki T (2008). Relation of dietary and other lifestyle traits to difference in serum adiponectin concentration of Japanese in Japan and Hawaii: the INTERLIPID study. AmJ Clin Nutr.

[40] Ostrowska L, Fiedorczuk J, Adamska E (2013). Effect of diet and other factors on serum adiponectin concentrations in patients with type 2 diabetes. Rocz Panstw Zakl Hig.

[41] Wu AH, Yu MC, Stanczyk FZ, Tseng C-C, Pike MC (2011). Anthropometric, dietary, and hormonal correlates of serum adiponectin in Asian American women. Nutr Cancer.

